# Interferon-stimulated gene of 20 kDa protein (ISG20) degrades RNA of hepatitis B virus to impede the replication of HBV *in vitro* and *in vivo*

**DOI:** 10.18632/oncotarget.11907

**Published:** 2016-09-08

**Authors:** Chean Ring Leong, Kenji Funami, Hiroyuki Oshiumi, Deng Mengao, Hiromi Takaki, Misako Matsumoto, Hussein H. Aly, Koichi Watashi, Kazuaki Chayama, Tsukasa Seya

**Affiliations:** ^1^ Department of Microbiology and Immunology, Graduate School of Medicine, Hokkaido University, Sapporo, 060-8638 Japan; ^2^ Department of Virology II, National Institute of Infectious Diseases, Tokyo, 162-8640 Japan; ^3^ Department of Gastroenterology and Metabolism, Applied Life Sciences, Institute of Biomedical and Health Sciences, Hiroshima University, Hiroshima, 734-8553 Japan; ^4^ Present address: Section of Bioengineering Technology, Universiti Kuala Lumpur (UNIKL) MICET, Kuala Lumpur, 78000 Malaysia; ^5^ Present address: Department of Immunology, Graduate School of Medical Science, Kumamoto University, Kumamoto, 860-8556 Japan

**Keywords:** hepatitis B virus (HBV), interferon (IFN), interferon-stimulated genes (ISGs), ISG20, gene therapy

## Abstract

Hepatitis B virus (HBV) barely induces host interferon (IFN)-stimulated genes (ISGs), which allows efficient HBV replication in the immortalized mouse hepatocytes as per human hepatocytes. Here we found that transfection of Isg20 plasmid robustly inhibits the HBV replication in HBV-infected hepatocytes irrespective of IRF3 or IFN promoter activation. Transfection of Isg20 is thus effective to eradicate HBV in the infected hepatocytes. Transfection of HBV genome or ε-stem of HBV pgRNA (active pgRNA moiety) failed to induce Isg20 in the hepatocytes, while control polyI:C (a viral dsRNA analogue mimic) activated MAVS pathway leading to production of type I IFN and then ISGsg20 via the IFN-α/β receptor (IFNAR). Consistently, addition of IFN-α induced Isg20 and partially suppressed HBV replication in hepatocytes. Chasing HBV RNA, DNA and proteins by blotting indicated that ISG20 expression decreased HBV RNA and replicative DNA in HBV-transfected cells, which resulted in low HBs antigen production and virus titer. The exonuclease domains of ISG20 mainly participated in HBV-RNA decay. *In vivo* hydrodynamic injection, ISG20 was crucial for suppressing HBV replication without degrading host RNA in the liver. Taken together, ISG20 acts as an innate anti-HBV effector that selectively degrades HBV RNA and blocks replication of infectious HBV particles. ISG20 would be a critical effector for ameliorating chronic HBV infection in the IFN therapy.

## INTRODUCTION

Hepatitis B virus (HBV) is a DNA virus having a unique life cycle consisting of DNA and RNA phases [1]. Type I interferon (IFN) appears effective at eradicating HBV in ∼30% of infected patients, but minimal type III and virtually no type I IFN can be induced in HBV-infected human hepatocytes [2]. The mechanisms for insufficient response of DNA/RNA sensors and IFN-mediated protection against HBV remain unclear.

Chronic infection with HBV accompanies covalently closed circular (ccc) DNA in the nucleus. Following cccDNA formation, viral mRNAs and pre-genomic (pg) RNA are transcribed [1]. The pgRNA is subsequently converted to a partially double-stranded genome by viral DNA polymerase. Unlike other DNA viruses, HBV uses an RNA proviral intermediate that must be copied back into DNA for replication [3]. Hence, both DNA and RNA sensors in the host cells can theoretically recognize HBV infection in human hepatocytes [4]. The DNA/RNA sensing system is conserved in mice and mouse hepatocytes permit HBV replication as in humans [4].

Mammals possess DNA/RNA-sensing pathways that activate a transcription factor, IRF3 to induce IFN-inducible genes via IFN-β and/or IFN-λ [4, 5]. The released IFNs engage with cell surface IFN receptors (IFNAR, IFNLR) that amplify IFN production via the JAK-STAT pathway. IFNAR consists of IFNaR1 and IFNaR2 while IFNLR of IL-28Ra and IL-10Rb [6]. Activation of IRF3 and STAT pathways induces the expression of hundreds of IFN-stimulated genes (ISGs) [5, 7]. Since the IFN-inducible genes may block the process of DNA/RNA amplification in hepatocytes during HBV replication, new antiviral strategies against CHB compensating for IFN therapy are being anticipated [5–7].

Although HBV has a strategy to circumvent host innate sensors by suppressing type I IFN induction [8], treatment of CHB patients with IFN suppresses viral replication to a certain extent. Ultimately, the host innate sensors stop viral replication through the recognition of replicative intermediate in both RNA and DNA phases. However, IFN-derived effectors that target the HBV life-cycle remain largely unexplored.

We screened a number of ISGs that may be involved in impeding HBV replication and found an anti-HBV activity in ISG20. ISG20 is an IFN-inducible 3′- to 5′-exonuclease that degrades DNA and RNA, as well as suppresses HBV antigen production in the hepatocytes. In a previous study, ISG20 is up-regulated for viral clearance in the liver of chimpanzees in response to infected HBV [9].

This study focused on the HBV-RNA-degrading function of ISG20 on the natural HBV replication system in immortalized mouse hepatocytes and the *in vivo* hydrodynamic injection model using IFNAR knockout mice. We demonstrated that ISG20 is one of the innate anti-HBV effectors of type I IFN output that is expected to be a target therapy for degradation of HBV RNAs.

## RESULTS

### IFN-β-induction in mouse hepatocytes correlates to the MAVS pathway

**Figure 1:**
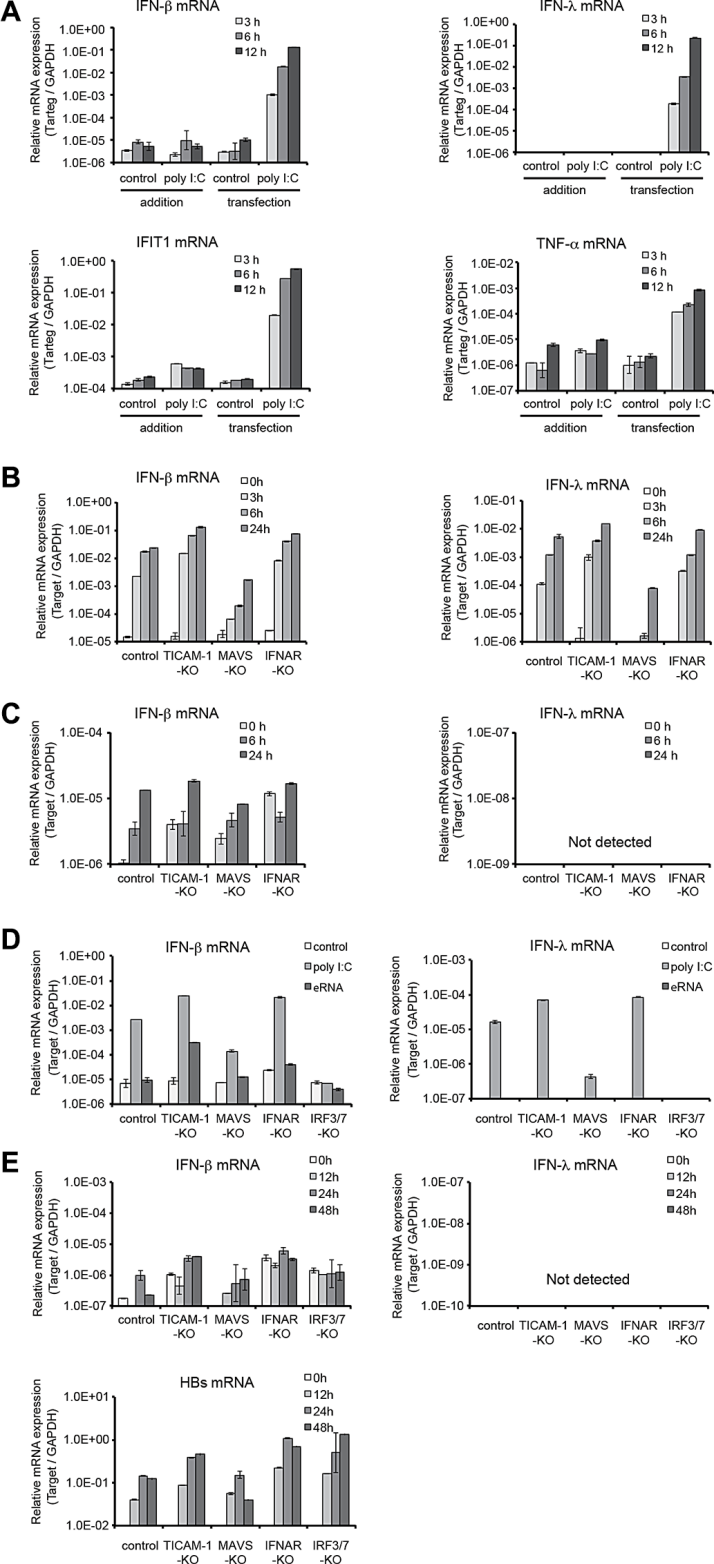
Interferon-inducible genes are expressed by MAVS-IFNAR pathway in immortalized mouse hepatocytes (**A**) Immortalized mouse hepatocytes were seeded to 24-well plate (1.5 × 10^5^/well) the day before stimulation. Poly I:C was directly added to medium (final 20 μg/ml) or transfected by lipofection (final 1.6 μg/ml) and cells were incubated for 3,6, or 12 hours. After stimulation, cells were harvested, total RNA was purified, and mRNA expression level was determined by quantitative RT-PCR. (**B**) Immortalized mouse hepatocytes generated established from *Ticam1*^−/−^, *Mavs*^−/−^, or *Ifnar*^−/−^ mice were seeded to 24-well plate the day before stimulation. Poly I:C transfection was performed as (A) and cells were incubated for 3, 6, or 24 hours. After transfection, mRNA quantification was performed as (A). (**C**) Immortalized mouse hepatocytes were stimulated with mouse IFN-α (final 2000 U/ml) for 6 or 24 hours. After stimulation, mRNA quantification was performed as (A). (**D**) Poly I:C or HBV εRNA was transfected to immortalized mouse hepatocytes by lipofection (final 1.6 μg/ml) and cells were incubated for 24 hours. After transfection, mRNA quantification was performed as (A). (**E**) The pTER1.4 × HBV plasmid was transfected to immortalized mouse hepatocytes from *Ticam1*^−/−^, *Mavs*^−/−^, *Ifnar*^−/−^, or *Irf3/7*^−/−^ mice by lipofection. Twenty-four hours after transfection, cells were harvested and mRNA quantification was performed as in (A).

We first examined the pathways involved in the induction of IFN-β and IFN–λ in immortalized mouse hepatocytes. Lipofection of polyI:C into the hepatocytes induced expression of IFN-β/λ mRNAs but not by the addition of polyI:C (Figure 1A). Only MAVS pathway was found responsible for the IFN-β/λ induction when the gene-disrupted hepatocytes were transfected with polyI:C (Figure 1B). Knockout of IFNAR barely affected the levels of IFN-β/λ mRNAs (Figure 1B). On the other hand, exogenous addition of IFN-α to these hepatocyte sublines induced uniformly low response levels of IFN-β mRNA expression irrespective of the gene disruption (Figure 1C). IFN-λ was not detected in all the sublines stimulated with IFN-α (Figure 1C). These observations are in good agreement with previous reports in which MAVS pathway in the cytoplasm signals the presence of dsRNA in the mouse hepatocyte sublines without involvement of TICAM-1 [10]. Type I IFN and IFNAR barely influence the inducible levels of IFN-β/λ mRNA. IFN-λ is not induced in response to IFN-α or beta in the hepatocytes.

Next we transfected the gene-disrupted hepatocyte sublines with ε-stem of HBV pgRNA, whilst1 PolyI:C was used as a control (Figure 1D). Upon polyI:C stimulation, IFN-β and -λ expression level was up-regulated in WT, TICAM-1^−/−^ and IFNAR^−/−^ hepatocytes with similar profiles. The IFN-β/λ expression was decreased about 100-fold in Mavs^−/−^ hepatocytes and abrogated completely in IRF3/7^−/−^ hepatocytes (Figure 1D). When these hepatocyte sublines were transfected with the ε–stem of HBV pgRNA, no up-regulation of IFN-β/λ expression was observed in all these sublines except TICAM1^−/−^ cells, where the IFN-β mRNA detected was about 10-fold higher than the WT hepatocytes (Figure 1D). In addition, we also transfected the hepatocyte sublines with a plasmid of the HBV full genome and found that IFN-β/λ expression level was not upregulated in any of the hepatocyte sublines (Figure 1E). The HBs mRNA, a product of the replicative HBV increased nearly one log in IFNAR^−/−^ and IRF3/7^−/−^ hepatocytes 48 h after the transfection with HBV plasmid (Figure 1E). In contrast, a lower expression level of HBs was detected in TICAM1^−/−^ hepatocytes at 24 and 48 h after the transfection. Thus, it can be concluded that HBV is well replicable in the IFNAR^−/−^ and IRF3/7^−/−^ hepatocytes and essentially no IFNs are principally produced in these hepatocytes during the infection. TICAM1^−/−^ hepatocytes show subtle susceptibility to HBV and permit mild induction of IFN-β. The reason for this controversial result is however remain unclear.

### IFN-inducible genes inhibit the HBs Ag production

**Figure 2:**
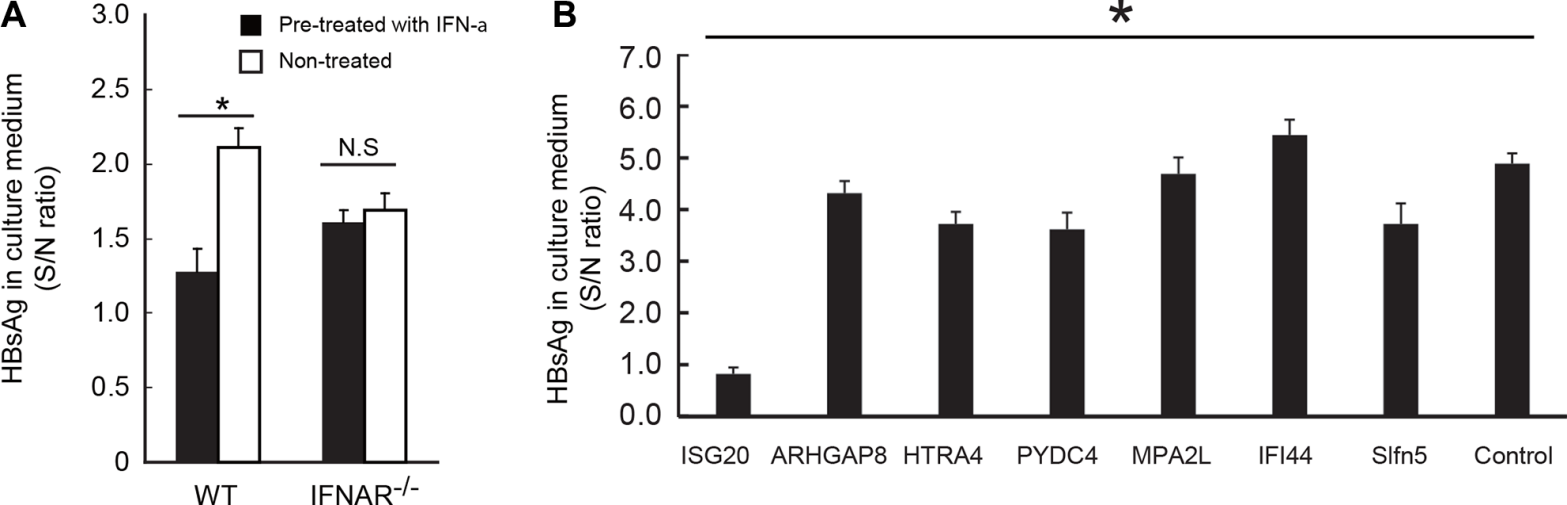
Identification of ISG20 as an inhibitor of HBV replication from interferon-inducible genes (**A**) Pre-treatment of IFN-α is crucial in suppressing HBV replication. The immortalized hepatocytes derived from the wild type and Ifnar−/− mice were pre-treated with 1000 unit of recombinant mus-IFN-α for 12 hours before transfected with the pTER1.4 × HBV. HBs antigen in the culture supernatant after 72 h post transfection was analyzed with ELISA. (**B**) Overexpressed ISG20 suppresses HBsAg production in HBV-transfected mouse hepatocytes. ISGs were cloned into the expression vector and co-transfected with the pTER1.4 × HBV into the wild-type immortalized hepatocytes to screen for the anti-HBV activity. HBs antigen in the culture supernatant after 72h post transfection was analyzed with ELISA. Data represent 3 independent experiments. *p < 0.05.×

The immortalized WT hepatocytes permitted HBs production when transfected with the plasmid of HBV full genome, and the HBs level in the medium was suppressed by pre-treatment of the cells with IFN-α (Figure 2A). This IFN-α-mediated blockage of HBs production was abrogated in IFNAR^−/−^ cells (Figure 2A), suggesting that some of the IFN-inducible rather than IRF3-inducible genes take part in the suppression of HBV replication. Therefore, we have chosen IFN-inducible genes from microarray DATA deposited to Gene Expression Omnibus, NCBI (Supplementary Table S1) to examine for their potential antiviral activity against HBV replication (Figure 2B). The IFN-inducible properties of these genes were confirmed in our microarray data (http://www.ncbi.nlm.nih.gov/geo/query/acc.cgi?acc=GSE75690). The selected genes were over-expressed in hepatocytes that were co-transfected with HBV plasmid, and the levels of HBs were determined by ELISA (Figure 2B). Of the genes tested, ISG20 strongly suppressed HBs production in the HBV-replicating mouse hepatocytes (Figure 2B). Similar results were obtained by ELISA using Huh7 cells transfected with HBV plasmid (Supplementary Figure S1A). Hence, we are able to demonstrate the HBV-suppressive function of ISG20 in hepatocytes-derived cell lines.

### ISG20 is barely induced by HBV or pgRNA stimulation in hepatocytes

**Figure 3:**
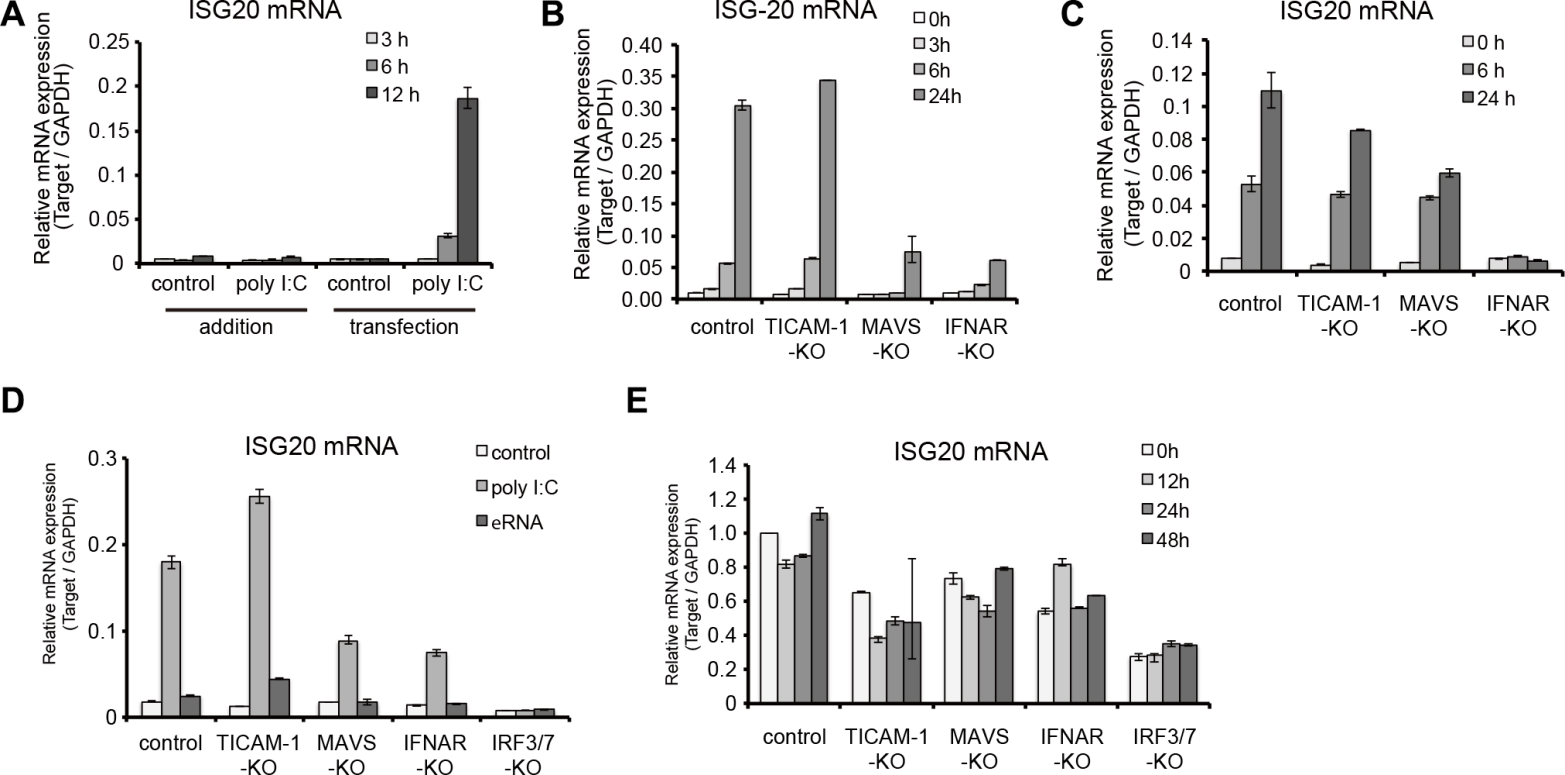
ISG20 is induced by type I IFN but not induced by HBV replication (**A**–**E**) All cDNAs were prepared as described in Figure 1(A–E), and ISG20 expression was determined by quantitative RT-PCR.

ISG20 was induced in mouse hepatocytes in response to the transfected polyI:C but such induction was barely detected in MAVS^−/−^ and IFNAR^−/−^ hepatocytes stimulated with polyI:C (Figure 3A and 3B). With the IFN-α treatment, ISG20 expression was up-regulated in WT, TICAM1^−/−^ and to a lesser extent MAVS^−/−^ hepatocytes. However, such exogenous additions of IFN-α did not lead to any increases of ISG20 expression in IFNAR^−/−^ hepatocytes (Figure 3C). We also transfected these hepatocyte sublines with the ε-stem of HBV pgRNA and plasmid of HBV full genome. ISG20 mRNA was up-regulated in MAVS^−/−^ and IFNAR^−/−^ hepatocytes when stimulated with pgRNA (ε-stem) (Figure 3D). On the contrary, up-regulation of the ISG20 expression was not observed in hepatocytes transfected with the plasmid of HBV full genome and parallels with the previous data that HBV full genome did not induce any production of IFN-β as well (Figure 3E). Thus, ISG20 is an IFN-inducible but not IRF3-inducible gene.

### ISG20 suppresses HBV replication in the hepatocytes-derived cell lines

**Figure 4:**
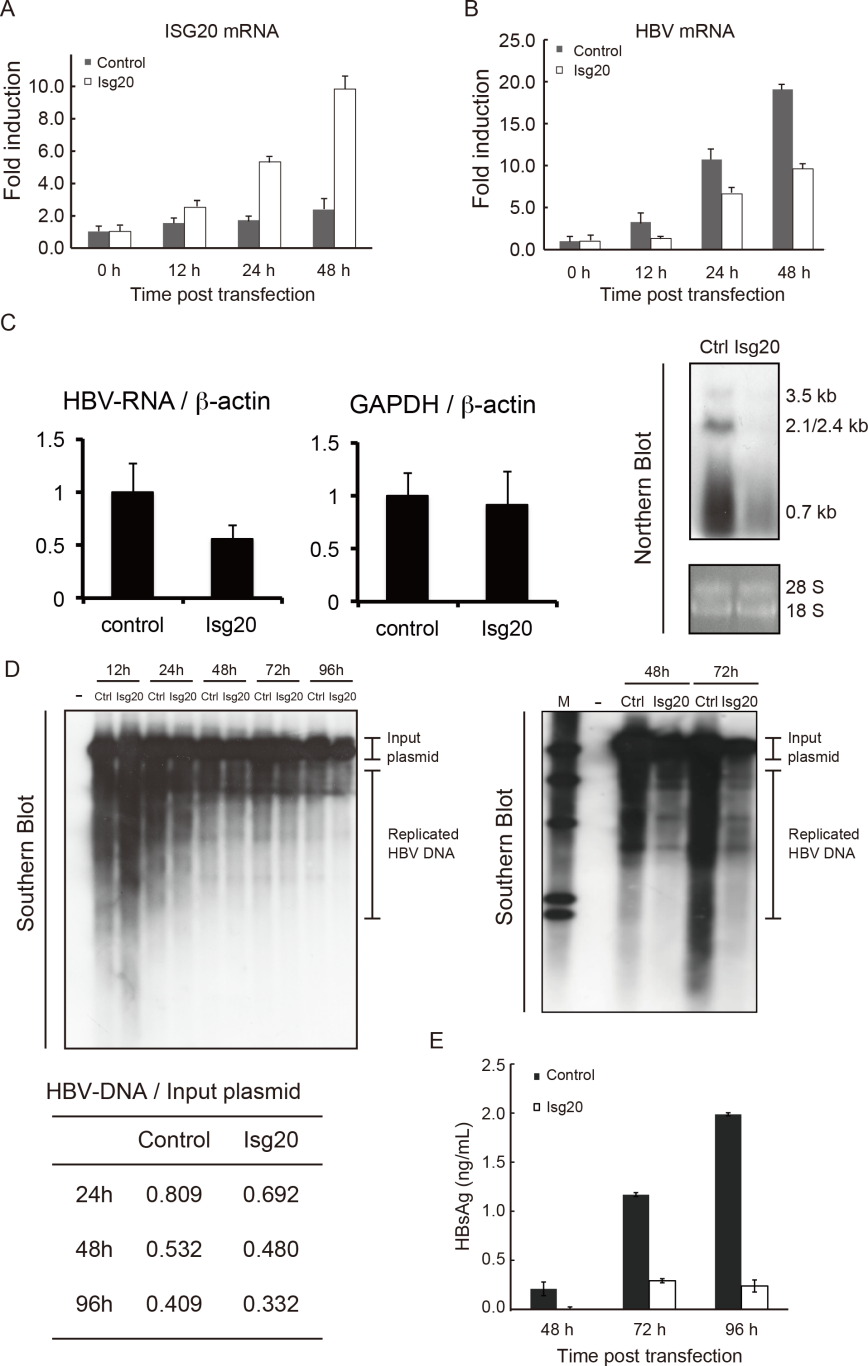
Inhibitory effects of ISG20 on HBV replication in cell culture (**A**) The immortalized mouse hepatocytes were cultured in the 24-well plates and co-transfected with 0.5 μg of Isg20 or control plasmid together with pTER1.4 × HBV. Total RNA was isolated at the time point post transfection and the expression of Isg20 mRNA was determined. (**B**) HBs mRNA were quantified with real-time RT-PCR under the same time-frame as panel A. (**C**) Similar experiment was repeated in Huh7 cells and 1 μg or 25 μg of the total cellular RNA was subjected to real-time RT-PCR or Northern blot analysis using the DIG-labelled probe specific to HBV, respectively. (**D**) The total cellular DNA was isolated 24 h, 48 h, 72 h, and 96 h after transfection and 500 ng of the isolated DNA was subjected to southern blot analysis using DIG-labelled probe specific to HBV (upper left and right figures). Cells with no pTER1.4 × HBV were used as a negative control (left lane in upper left and right figures). Relative values of replicated HBV DNA were quantified using upper left figure and showed at lower figure. Data represent 3 independent experiments. (**E**) The culture supernatant of hepatocytes transfected with Isg20 or control plasmid was collected at the indicated time points. The levels of HBs Ag were analyzed with ELISA.

We then overexpressed ISG20 in WT hepatocytes. The mRNA of ISG20 increased up to 48 h after the transfection (Figure 4A). In order to examine ISG20 for its potential antiviral activity against HBV replication, ISG20 was co-transfected with HBV full genome. The levels of HBV mRNA were suppressed in hepatocytes transfected with ISG20 compared to the control (Figure 4B).

In human HepG2 cells transfected with Isg20 and HBV plasmids, HBs production started increasing around 24 h and ISG20 mRNA was elevated concomitant with the blocking of HBs production (Supplementary Figure S1B and S1C). These results were similar to the results obtained with mouse hepatocytes transfected with Isg20 and HBV plasmids (Figure 4A and 4B). Thus, ISG20 ectopically expressed in mouse hepatocytes extensively suppressed HBs products after the expression of Isg20.

We further investigated the mechanisms of HBV inhibition by ISG20. We used Huh7 cells for this purpose to detect substantially high levels of HBV DNA/RNA signal. Total cellular RNA from HBV-plasmid-transfected cells was analyzed with Northern blots using an HBV-specific probe. A significant reduction in HBV RNAs of 3.5 kb, 2.4/2.1 kb and 0.7 kb was observed (Figure 4C, right figure). In the same ISG20-overexpressed cells, HBV-RNAs were specifically reduced with reference to the control host RNAs (Figure 4C, left and center figures). Reduction in steady-state levels of viral pgRNA, which is the template for HBV DNA synthesis, could be the primary cause of HBV replication suppression in Huh7 cells. However, the levels of the 2.4/2.1 kb and 0.7 kb HBV mRNA, which showed 100% sequence identity to the partial pgRNA, were also decreased in response to ISG20 expression.

Since ISG20 is a 3′-to-5′ exonuclease *in vitro* with a lesser specificity to DNA [11, 12], we needed to rule out the possibility that ISG20 suppressed HBV replication by altering the stability of HBV transcription templates directly by degrading transfected HBV plasmids. ISG20 overexpression reduced HBV DNA replication levels as detected by Southern blots (Figure 4D, upper left and upper right figure). Examination of the co-transfected HBV plasmids or input plasmid signals, detected by *Pst*I digestion and DNA hybridization (∼9.7 kbp), showed that the input plasmid were almost unaffected in response to ISG20 overexpression, although the replicative HBV DNAs, relatively quantified as the ratio between plasmid and replicative HBV-DNA, were significantly decreased compared to controls (Figure 4D, lower figure). Consequently, down-regulated HBV RNA and replicative DNA mediated by the ISG20 led to a reduction in HBs antigen during viral replication in Huh7 cell culture supernatant (Figure 4E). These results suggest that ISG20 antiviral function is a host factor in controlling the intracellular HBV replication cycle.

### ISG20 post-transcriptionally regulates the steady state of HBV RNA

**Figure 5:**
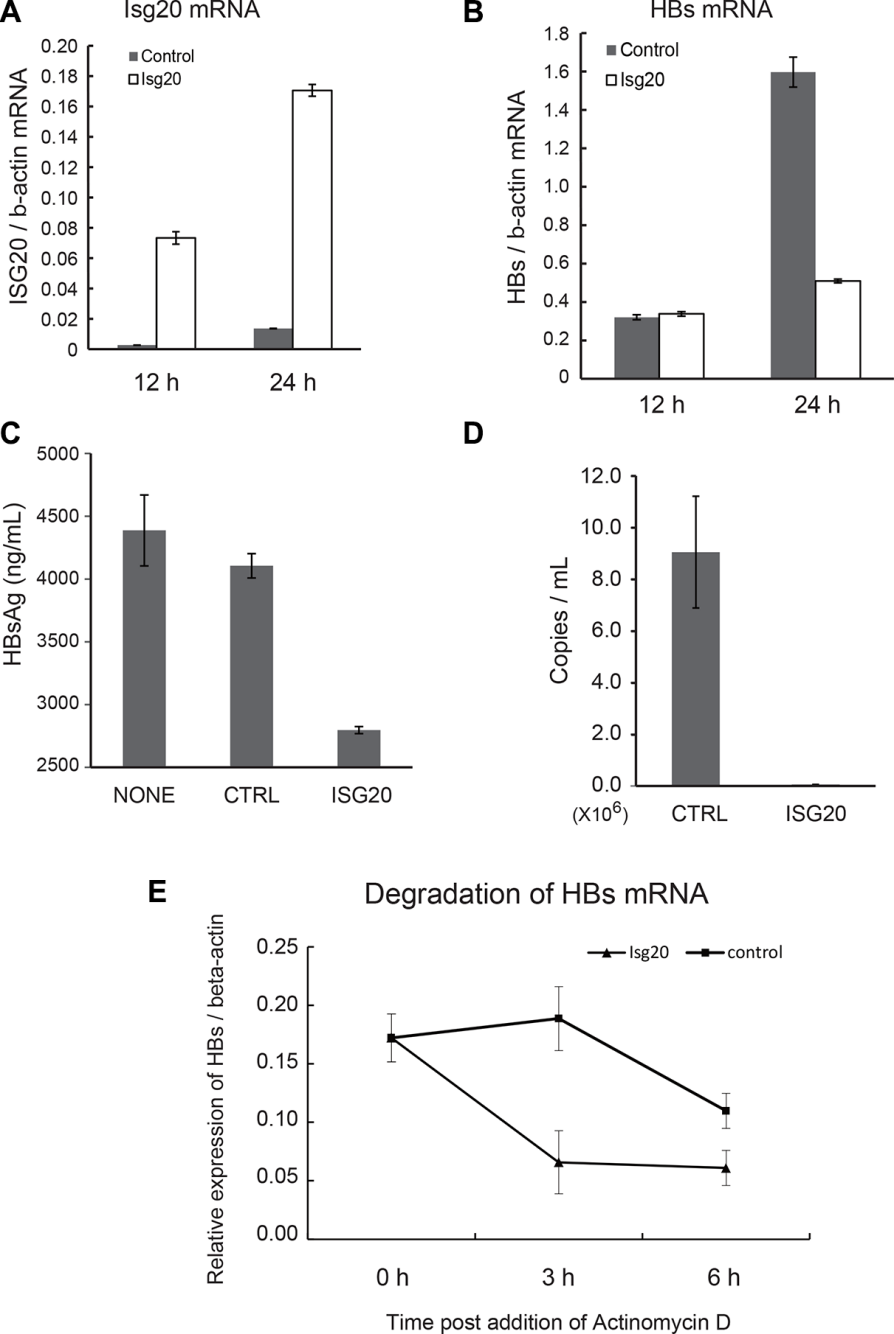
ISG20 down-regulates HBV RNA via posttranscriptional mechanism (**A**) ISG20 level. HBV-T23 cells that constitutively produces the HBV virion were transfected with the control plasmid or *Isg20*-expressing plasmid, and the levels of *Isg20* were measured by qPCR at 12 and 24 h. (**B**) The levels of HBs mRNA was determined by qPCR at 12 and 24 h post transfection. (**C**) Culture supernatant was collected after 24 h post transfection with *Isg20*-expressing plasmid and HBs Ag was determined by ELISA. (**D**) In the same samples as panel B, the DNA content of the virions in the culture supernatant reflecting the HBV copies was determined by qPCR. (**E**) ISG20 HBV-regulatory function is apart from transcription. Actinomycin D was used as a transcription inhibitor for the addition to the HBV-T23 cells that had been transfected with the control or ISG20 expression plasmid. Total RNA was isolated at the indicated time point and HBs mRNA was quantified with real-time RT-PCR.

To determine if ISG20-mediated down-regulation of HBV RNA was due to a transcriptional or posttranscriptional mechanism, we overexpressed ISG20 in the HBV-stable cell line HBV-T23, in which HBV RNA is transcribed from an integrated transgene [13]. ISG20 was expressed in transfected cells at 12 h and 24 h (Figure 5A). ISG20-mediated suppression of the HBs mRNA was detected in the HBV-T23 cells at 24 h (Figure 5B). At this time point after the transfection, HBs Ag levels (Figure 5C) and product DNA levels (Figure 5D) were also decreased in the culture supernatant. ISG20-mediated down-regulation of HBV replication is a post-transcriptional mechanism.

To further endorse the above finding, we directly measured decay kinetics of HBV RNA upon ISG20 overexpression in the presence or absence of actinomycin D. HBV-T23 cells were transfected with control or ISG20 expression vector for 24 h before the addition of actinomycin D, a compound that non-specifically inhibits transcription by forming a stable complex with double-stranded DNA via deoxyguanosine residues. This inhibits DNA-primed RNA synthesis from HBV DNA. The HBV RNA was quantified with real-time RT-PCR in a time-course study. HBV RNA degradation was faster in the presence of overexpressed ISG20 than in the control vector (Figure 5E), suggesting that ISG20 promotes HBV RNA degradation.

### ISG20′s Exo domains sustain anti-HBV function

ISG20 belongs to the DEDD superfamily and contains three separate exonuclease domain (Exo I-III) motifs that are highly conserved among rat, mouse and human ISG20 [11, 14]. The conformation and spatial disposition of ISG20 are known from the crystal structure of human ISG20 [14].

**Figure 6:**
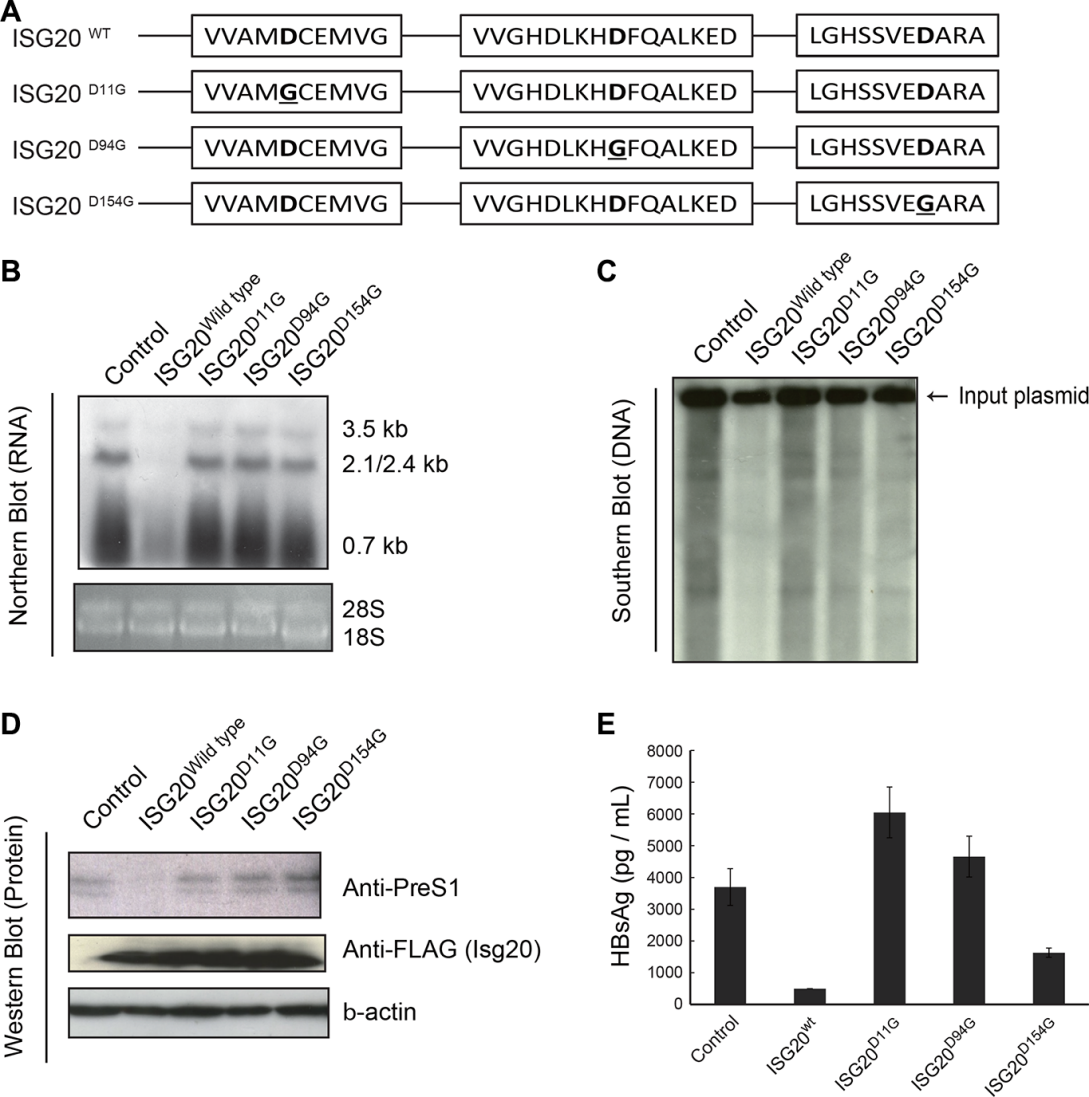
Mapping the exonuclease domain of Isg20 on the HBV antiviral activity (**A**) Schematic structure of the ISG20 with the three exonuclease domains. Mutations that disrupt each individual Exo domain are indicated as superscript according to previous studies [11]. (**B**) Huh7 cells were co-transfected with pTER1.4HBV and the wild type ISG20 or its mutants. Total RNA was isolated from the cells at 48 h post transfection and the RNA (25 μg) was subjected to Northern blot using the HBV specific probe. (**C**) The cellular DNA of Huh 7 cells co-transfected with the pTER1.4 × HBV and ISG20 or its mutant(s) expression plasmid was isolated and subjected to Southern blot analysis using DIG-labelled probe specific to the HBV. (**D**) Western blot was performed for protein analysis of Huh7 cells with pTER1.4HBV and the wild type ISG20 or its mutants. Proteins were detected with anti-FLAG and anti-Pre-S1 antibodies. Beta-actin served as a protein loading control. (**E**) HBsAg in the culture supernatants was analyzed with ELISA. The culture supernatant of Huh7 cells transfected with pTER1.4HBV and the wild type ISG20 or its mutants were harvested 48 h after the transfection. The HBs Ag levels were determined by ELISA.

Asp11, Asp94 and Asp 154 in the Exo I, II and III domains are active centers for ISG20 exonuclease [11, 12]. To determine the requirement of each exonuclease domain for ISG20-mediated HBV suppression, we introduced amino acid mutations into the Exo domains to disrupt the active site structure based on previous studies (Figure 6A). The mutant ISG20 proteins were co-expressed with HBV in Huh7 cells and effects on viral RNA (Figure 6B), DNA (Figure 6C) and protein (Figure 6D) were analyzed. Disruption of each individual Exo domain, particularly ISG20-D11G (Exo I) or ISG20-D94G (Exo II), abolished ISG20-mediated HBV RNA decay (Figure 6B) and led to high production of HBs antigen in the culture supernatant of transfected Huh7 cells (Figure 6E). Mutation of the Exo III domain (ISG20-D154G) only partially decreased the antiviral activity of ISG20 concomitant with viral genome or RNA degradation (Figure 6B, 6C). Although protein expression appeared similar in ISG20-transfected and ISG20 mutant-transfected Huh7 cells, only wild-type ISG20-transfected cells failed to produce PreS1 protein (Figure 6D). These observations indicate that the exonuclease activity of ISG20 is indispensable for HBV-suppressing activity of ISG20. The Asp residues in the exonuclease-active domains I and II contribute to the anti-HBV function of ISG20, though the Asp in the Exo III domain little participates in the optimal antiviral activity of ISG20.

### ISG20 is crucial for *in vivo* suppression of HBV replication in Ifnar^−/−^ mice

**Figure 7:**
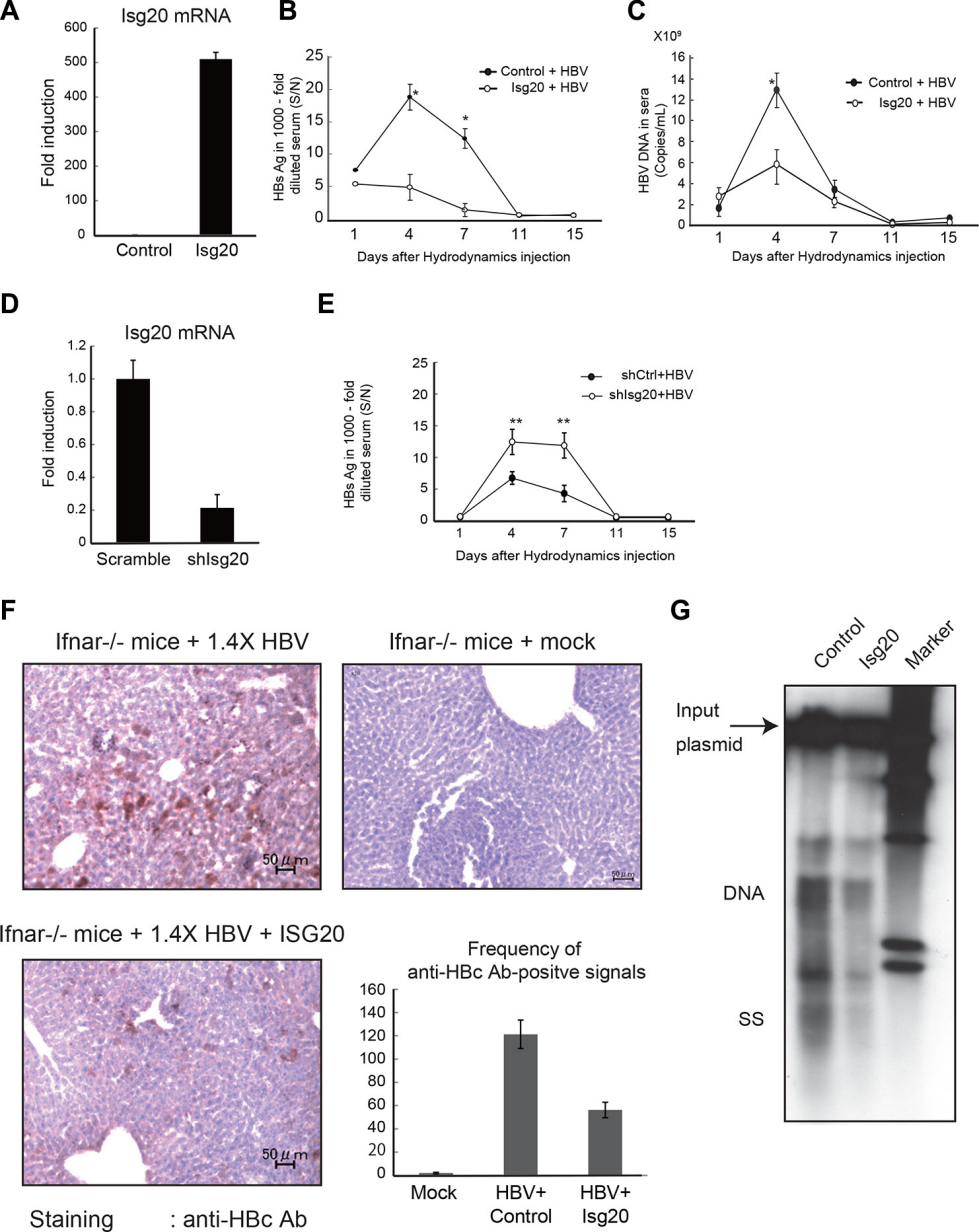
ISG20 suppresses HBV replication in Ifnar^−/−^ mice liver (**A**) The *Ifnar^−/−^* mice were hydrodynamically injected with 50 μg of the pTER-1.4 × HBV plasmid together with the control or ISG20 plasmid. Total RNA was isolated from liver on day 3 post injection and determined the mRNA levels of ISG20 by qRT-PCR. (**B**) HBsAg was measured with sera isolated at the indicated time points from *Ifnar^−/−^* mice injected with the plasmid as in panel A. The HBsAg titers were determined with 1000-fold diluted sea. (●), mice receiving the control plasmid; (○), mice receiving the ISG20 plasmid. (**C**) The serum HBsAg titers were determined with an enzyme immunoassay at O.D 450 nm [calculated as signal over noise ratios (S/N)]. HBV DNA in the sera was measured as in panel B. The levels of HBV DNA were determined by qPCR and indicated as copies per mL. Error bars indicate SD here and in the other figures. (**D**) Knockdown of *Isg20* by injection of shRNA in the mouse liver. Hydrodynamic injection was performed with the plasmid containing shRNA for *Isg20* (20 μg per mice). After 3 days, the total RNA was isolated from the liver as in Panel A, and the levels of I*sg20* were determined by qPCR. A scrambled RNA was used as a control. (**E**) Similar study to panel B and C was conducted in the wild-type mice using the pTER-1.4 × HBV and the plasmid of *Isg20* shRNA to silence the endogenous ISG20. The scrambled RNA was the control for the shRNA. (**F**) Histochemical analysis of the *Ifnar^−/−^* mice. The mice were hydrodynamically injected with 20 μg of the pTER1.4 × HBV plasmid together with the control (right upper panel) of ISG20 expression plasmid (left upper panel). HBc protein in the livers on day 3 post injection were visualized with immunohistochemical staining of the mouse liver sections embedded in the OCT compound using an anti-HBc antibody for the detection of HBcAg. Representative sections are shown. HBcAg-positive cells in the *Ifnar^−/−^* mice receiving the ISG20 expression plasmid (upper panel) or those with control plasmid (lower panel). The scale bars represent 10 μm. The images are displayed at 200× magnification. Frequency of HBcAg-positive signals between the different mouse strains shown is based on the 3 images of each (right lower panel). (**G**) On day 3 post injection, the mice were sacrificed and liver DNA was extracted. The extracted DNA was analyzed with Southern blot using an HBV-specific probe. The sizes corresponding to the injected DNA (arrow) and the replicative intermediates (DNA, ss) are indicated to the left. M, marker.

Since immortalized *Ifnar^−/−^* hepatocytes we previously established [15] were more permissive to HBV replication via plasmid transfection than WT hepatocytes, we hypothesized that type I IFN and IFNAR were required to suppress HBV replication in mouse hepatocytes *in vivo*. We tested if HBV replication in *Ifnar^−/−^* mice was blocked by simultaneous administration of Isg20 plasmid to livers *in vivo*. We used hydrodynamic injection to introduce replication-competent full HBV genomes into the livers of *Ifnar^−/−^* mice [10]. HBV DNA reaches the nucleus of hepatocytes in mouse livers, where it is transcribed into viral transcripts and rapidly translated to produce high serum titers of HBs, HBc and Dane particles [15]. When we hydrodynamically injected HBV DNA and Isg20 plasmid, Isg20 was highly expressed in the liver (Figure 7A), indicating that hydrodynamically injected genes and HBV plasmid were predominantly expressed in the liver. Based on these results, we examined the *in vivo* effectiveness of ISG20 in *Ifnar^−/−^* mice by hydrodynamically injecting the *Isg20* expression plasmid together with the full HBV genome. *Isg20* was expressed in mouse livers that received the plasmid.

A time-course of HBV DNA and HBs Ag in the sera of *Ifnar*^−/−^ mice is shown in Figure 7B and 7C. HBs antigen and HBV DNA in the serum of the *Ifnar^−/−^* mice were highly elevated at day 4 and 7 post injection compared to mice receiving the control plasmid. In contrast, robust viral replication at an early time point was abrogated when the Isg20 was overexpressed in mouse livers. Similar experiments were conducted using WT mice to investigate the effect on HBV replication of silencing the intrahepatic endogenous Isg20 (Figure 7D and 7E). Endogenous Isg20 was abolished in mice using specific but not non-specific siRNA (Figure 7D). Reduced Isg20 with siRNA resulted in higher HBs antigen in mouse serum compared to the control. These results indicate that ISG20 is a crucial molecule for IFN-stimulated genes that suppress HBV replication *in vivo*.

The *Ifnar^−/−^* mice transfected with the HBV full genome and control or Isg20 plasmid exhibited different HBc Ag expression in livers (Figure 7F). The frequency of the HBc Ag-positive hepatocytes was decreased in mouse livers ectopically expressing Isg20 (Figure 7F), consistent with results from intrahepatic viral replication and serum antigenemia. We also examined intrahepatic viral replication in mouse livers post injection. Southern blots using total DNA from mouse livers on day 3 post injection (Figure 7G) showed that ISG20 overexpression did not have a notable effect on the input plasmid but a more significant impact was observed on HBV replicative intermediates (Figure 7G).

### ISG20 inhibits the susceptibility of NTCP-HepG2 to HBV

**Figure 8:**
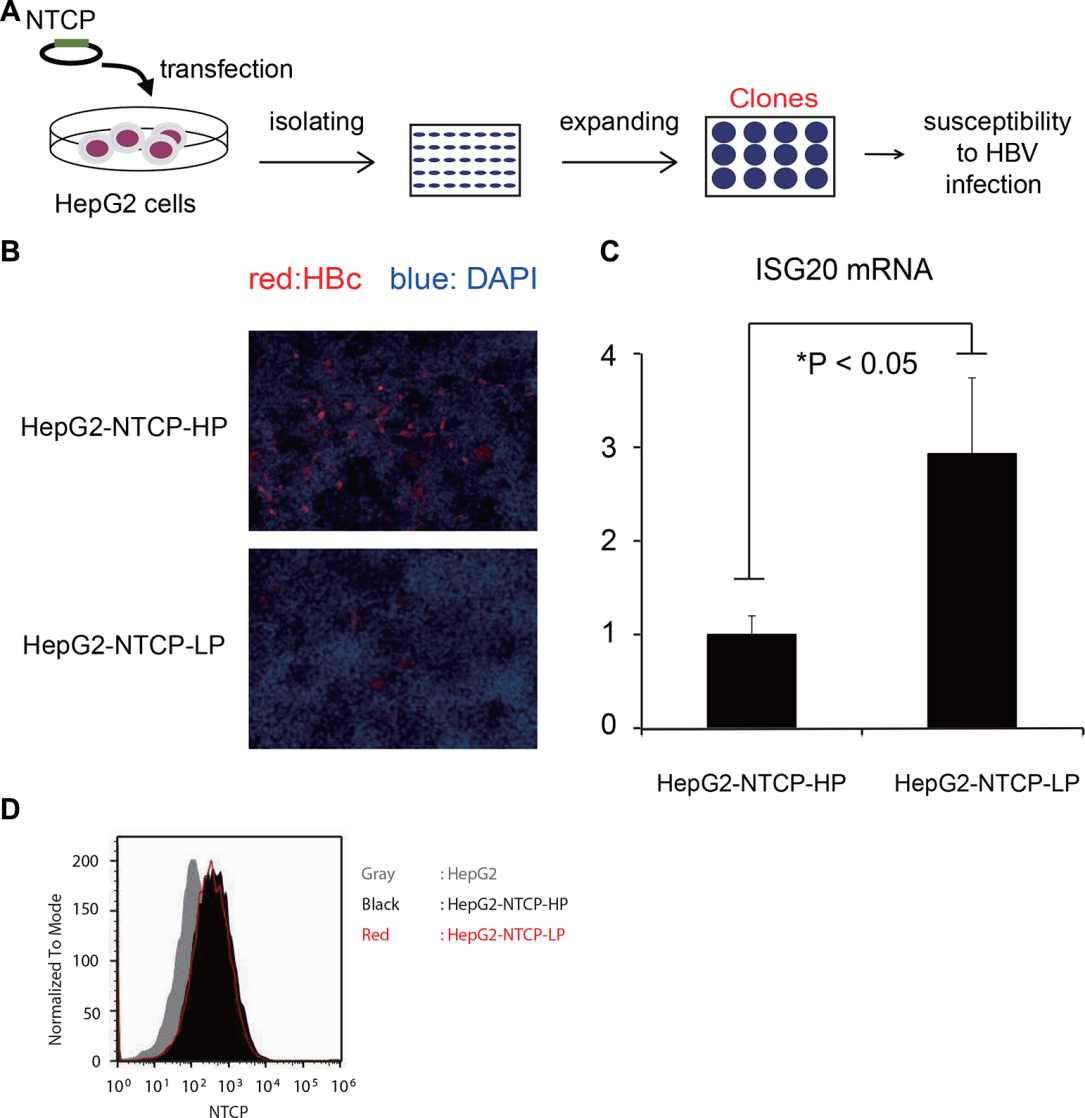
ISG20 expression is crucial in the HBV susceptibility (**A**) Schematic illustration of the establishment of HepG2 clones that constitutively express the entry receptor of HBV, NTCP as described previously [18]. (**B**) The susceptibility of these clones to HBV was examined by imaging analysis. Two HepG2 clones overexpressing NTCP (HepG2-NTCP-HP and -LP) were incubated with HBV for 16 h and detected for intracellular HBc protein at 12 days post-infection by immunofluorescence. The panel a top shows a clone that was highly susceptible while the panel at the bottom shows the clone with lower susceptibility to HBV. Red and blue signals indicate HBc protein and nucleus, respectively. NTCP protein on the cell surface was probed with a fluorescence-labeled preS1-peptide (2–48 a.a). Red, black and gray indicate HepG2-NTCP-LP, HepG2-NTCP-HP and parental HepG2 cells, respectively. (**C**) The endogenous expression levels of ISG20 in the two clones were determined by qPCR. (**D**) The expression levels of NTCP in the two clones were checked by flow cytometry.

Recently, the sodium taurocholate cotransporting polypeptide (NTCP) membrane transporter was reported as an HBV entry receptor [16, 17]. We engineered a strain of HepG2 cells to overexpress the NTCP gene to produce HBV-permissive cell lines (Figure 8A) [18]. Two of these clones that expressed an equivalent level of cell-surface NTCP protein showed different permissiveness to HBV [HepG2-NTCP-high permissive (HP) and HepG2-NTCP-low permissive (LP) cells] (Figure 8B). To investigate host factors leading to differences in viral permissiveness, we analyzed endogenous expression of ISG20 in these two different clones and found that ISG20 expression was about 80% lower in the clone susceptible to HBV infection (Figure 8C), despite their similar NTCP expression levels (Figure 8D). Thus, low endogenous expression of ISG20 might be associated with host cell permissiveness to HBV.

## DISCUSSION

IFN-inducible RNases, RNaseL and ISG20, cooperatively act for immediate eradication of viruses. [12, 19]. ISG20 inhibits replication of various RNA viruses [11, 20]. However, solid *in vivo* evidence on its antiviral properties has not been reported on a DNA virus HBV. Type I and III IFNs induce ISG20, but HBV infection does not facilitate IFN induction, thereby no ISG20 functioning for HBV eradication in human and mouse hepatocytes.

Here we demonstrated that overexpression of Isg20 impeded the replication of HBV in a mouse hepatocyte system. *In vivo* transfection studies employing the hydrodynamic injection method shows that HBV replication was more progressive in *Ifnar^−/−^* mice than in WT mice. Furthermore, simultaneous introduction of Isg20 and HBV plasmid into the *Ifnar^−/−^* mice suppressed HBV replication. In fact, the increase of ISG20 expression paralleled the suppression of HBs production in the liver of *Ifnar^−/−^* mice injected with HBV plasmid and Isg20. In NTCP-transfected HepG2 studies [18], low HBV susceptibility was attributable to high expression of ISG20; the result appears to be on-target. Several clinical studies suggest that high ISG20 level is associated with positive response to IFN-α treatment in CHB patients [21, 22]. Our current study supports the correlation between IFN responders and incremental ISG20 mRNA level in CHB patients [23].

However, ISG20 was hardly induced by the IRF3-activating genes via MAVS or TICAM-1 pathways in the mouse hepatocytes transfected with HBV- or ε– RNA. ISG20 inhibits HBV replication by downregulating HBV RNA (both viral mRNA and pgRNA) as well as the viral replicative DNA without degrading the host 18S and 28S rRNA. If the type I IFN-IFNAR axis induces Isg20, it is therefore crucial for the early phase protection of hepatocytes against HBV as demonstrated in the mice *in vivo* transfection studies. Because HBV replication barely activates the IFN-inducing or MAVS pathway while, the way for direct induction of ISG20 as well as IFN-(β/λ) is an interesting therapy for HBV-infected patients.

RNase L is a prominent IFN-inducible RNase that is critical for evoking the innate antiviral response. RNase L is localized to ‘stress granule’ near mitochondria [23], while ISG20 is mainly found in the nucleolus and the Cajal bodies [24, 25]. In earlier studies, RNase L degrades non-self (viral) RNA [11, 20], but recent analysis suggests that RNase L sequesters even self RNA and the fragmented RNAs are recognized by RIG-I and MDA-5 [26]. Hence, RNase L degrades viral RNAs along with cellular 18 S and 28 S rRNAs, which leads to infected cell apoptosis to prevent viral spreading. ISG20 essentially differs from RNase L for HBV targeting in that its exonuclease function specifically directs only to viral RNA/DNA [26]. Our results also suggest that ISG20 degrades HBV RNA more rapidly than HBV DNA including the replicative DNA in the HBV life cycle. The two different modes of nucleases may cooperatively act to eliminate HBV DNA and pgRNA during IFN therapies in CHB patients [22, 27].

HBV RNAs possess a unique stem-loop structure at the 5′- end of the pgRNA, called the epsilon element (ε) [28]. The ε-stem loop is vital for the HBV life cycle since reverse transcription of the virus RNA requires recognition by the ε-stem to trigger assembly of the nucleocapsid [28]. The stem-loop structure might recruit nucleases including ISG20, similar to the stem-coupling nucleases that tune transcript stability [29, 30]. However, the ε-stem of pgRNA *per se* is unlikely to recruit other intrinsic host proteins to ISG20 to suppress the HBV replication cycle. Direct expression of Isg20 may be a better strategy than ε-stem introduction to specifically degrade HBV DNA/RNA.

We previously found that STING predominantly participates as a DNA sensor in HBV-DNA recognition in mouse cells [4]. RIG-I/MDA5 and MAVS facilitate IRF3 activation in human but not mouse hepatocytes [4, 10]. Hence, our results are, in part, incompatible with previous reports on human studies, which may reflect the difference of species- and cell type-specific expression profiles of IFN-related genes. Whether the HBV receptors including NTCP work in conjunction with antiviral signaling including DNA-sensing pathways is intriguing as a future study. Although we do not know by what degrees DNA-sensing pathways participate in inhibiting HBV replication in hepatocytes, non-parenchymal cells appear to be source of the IFN for induction of hepatocyte ISG20 (Funami, Seya et al., unpublished data). Since therapeutic type I IFN up-regulates the potential of the MAVS pathway in stromal cells, HBV DNA or RNA (including mRNA and pgRNA) may act on stromal cells to further liberate type I IFN, that in turn up-regulate ISG20 in hepatocytes. Yet, we have not identified the route of stroma cell stimulation either indirectly (via exosomes released from HBV-infected hepatocytes) or directly (via HBV plasmid) [31].

The chimpanzee studies show that most genes up-regulated by HBV are IFN-γ-inducible genes, implicating cellular immunity involving T and NK cells in the protective response against HBV infection [32, 33]. Consistently, IFN-γ is highly up-regulated in a mouse hydrodynamic injection model of HBV [10]. Activated NK cells and T cells are major sources of IFN-γ and actively take part in eliminating HBV-infected cells [34, 35]. IFN-γ in cellular immunity induces Isg20 and other antiviral genes including Ddx60 in hepatocytes. DDX60 is a RNA helicase that acts as an agent of the RNA exosome, which may act for eliminating viral RNA [36, 37]. Other IFN-inducible genes would contribute to network formation from innate to cellular immune activation in microenvironment [38, 39].

In conclusion, early phase IFN-inducible nucleases and late phase cellular effectors participate in HBV regulation. ISG20 is induced by IFNs and targets HBV RNAs to eliminate early phase of HBV throughout early and late phases. The function of ISG20 in conjunction with the cellular immune system needs to be further characterized in HBV patients.

## MATERIALS AND METHODS

### Mouse and hepatocytes

All mice were backcrossed with C57BL/6 mice more than seven times before use. *Irf3/7*^−/−^ and *Ifnar*^−/−^ mice were kindly provided by T. Taniguchi (University of Tokyo, Tokyo, Japan). *Ticam1*^−/−^ and *Mavs-1*^−/−^ mice were established in our laboratory as described previously [24, 40, 41]. *Irf3*^−/−^ and *Irf7*^−/−^ mice were generated from a single backcross of *Irf3/7*^−/−^ double knockout mice in our laboratory. Female C57BL/6J mice were purchased from Japan Clea (Tokyo) and used at 7–9 weeks of age. All mice were maintained under specific pathogen-free conditions in the Animal Facility at Hokkaido University Graduate School of Medicine (Sapporo, Japan). Animal experiments were performed according to the guidelines set by the animal safety center, Hokkaido University, Japan.

Mouse immortalized hepatocytes were established from *Ifnar*^−/−^, *Irf3/7*^−/−^, *Mavs*^−/−^, *Ticam1^−/−^* mice in our previous report [15]. Hepatocytes were cultured in high-glucose Dulbecco's modified Eagle's medium (DMEM; Invitrogen, Tokyo, Japan) supplemented with 2 mM L-glutamine, 100 U of penicillin/ml, 100 μg of streptomycin/ml, 10% fetal bovine serum, 20 mM HEPES (Invitrogen), 30 μg/mL L-proline, 0.5 μg/mL insulin (Sigma, St. Louis, MO, USA), 100 pM dexamethasone (Wako, Osaka, Japan), 44 mM NaHCO_3_, 10 mM nicotinamide (Wako), 10 ng/mL EGF (Wako), 0.2 mM L-ascorbic acid 2-phosphate (Wako), and 1% MEM-non essential amino acids (Invitrogen).

### Animal studies with hydrodynamic injection of HBV full genome plasmid

A total of 50 μg of the HBV plasmid [42] was injected into the tail vein of mice in a volume of 2.0 ml TransIT-QR hydrodynamic delivery solution (Mirus, USA) [10]. In some experiments, *Isg20* plasmid or siRNA was co-injected with HBV plasmid. Plasmid DNA was prepared by using an EndoFree plasmid system (Qiagen, Germany) according to manufacturer's instructions. The endotoxin-free quality was checked by Limulus test with all DNAs before use [43]. The total volume was delivered within 3–8 seconds as described [10].

### Cell cultures and plasmid

Human hepatocytes-derived HepG2 and Huh7 cells were obtained from the ATCC and maintained in DMEM medium supplemented with 10% fetal bovine serum, 100 U/mL penicillin and 100 μg/mL streptomycin [4, 44]. The HBV119 T23 is a human hepatocyte-derived cell line that constitutively produces the HBV virions [13]; the cells were cultured in the DMEM with the addition of hygromycin B to maintain the HBV genome-integrated cells. Recombinant mouse IFN-α was purchased from Sigma-Aldrich. The pTER1.4 × HBV plasmid containing 1.4 genome length sequences of HBV was used in this study to produce a similar sedimentation in sucrose density gradient centrifugation to HBV extracted from the serum of HBV carriers [13]. The human Isg20 was cloned into the plasmid with a CMV-IE promoter using the specific primers. Disruption of the individual exonuclease domain in the Isg20 cDNA was carried out using QuickChange II site-Directed mutagenesis kit (Agilent). All of the above wild-type and mutant Isg20 genes have an N-terminal Flag-tag sequence in the expression vectors.

### Northern blot assay

Total cellular RNA including viral RNA was isolated with Trizol and 25 μg of the isolated RNA was resolved in a 1.5% agarose gel containing 2.2 M formaldehyde. The RNA in the gel was transferred onto IMMOBILON NY+ charged nylon membrane (Milipore) for Northern blot analysis. RNAs on the sheet were detected with specific DIG-labeled probe using a full-length HBV-DNA template as described previously [45]. The RNA was detected with the DIG DNA labeling and detection kit (Roche Diagnostics, Basel, Switzerland) according to the instructions. The levels of reference gene mRNAs in total RNA were quantified by quantitative PCR as described below.

### Southern blot assay

Viral DNA was isolated from intracellular viral capsids and detected with specific DIG-labeled probe as described previously [10]. In brief, to isolate the viral DNA, homogenized mouse livers or cells transfected with the HBV plasmid were subjected to an overnight sodium dodecyl sulfate-proteinase K digestion followed by phenol extraction and ethanol precipitation. 20 μg of the isolated DNA was separated in a 1% agarose gel, transferred onto IMMOBILON NY+ charged nylon membrane (Milipore) and detected with a HBV-specific DIG-labeled probe using a full-length HBV-DNA template. HBV-DNA-specific signals were quantified using Image J, and plasmid DNA signal was used as reference.

### Western blot assay

Cells were lysed with RIPA buffer and separated on a 10% SDS-PAGE. Proteins were transferred onto Immobilon PVDF-FL membrane (Milipore). The membranes were blocked with 3% skim milk and probed with antibodies against FLAG-tag, HBc antigen, or β-actin. Bound antibodies were visualized with HRP-bound secondary antibodies. The immunoblotting signals were quantified as reported [46].

### HBV preparation and infection

HepG2 and HepG2-NTCP-HP and HepG2-NTCP-LP cells that constitutively express the sodium taurocholate co-transporting polypeptide (NTCP) membrane transporter were cultured with DMEM/F-12 + GlutaMax (Invitrogen) supplemented with 10 mM HEPES (Invitrogen), 200 units/ml penicillin, 200 μg/ml streptomycin, 10% FBS, 50 μM hydrocortisone and 5 μg/ml insulin in the presence (HepG2-NTCP-HP and –LP cells) or absence (HepG2 cells) of 400 μg/ml G418 (Nacalai) as reported previously [18]. NTCP-expressing HepG2 cells were infected with HBV at 6,000 genome equivalent (GEq)/cell.

HepG2, HepG2-NTCP-HP or HepG2-NTCP-LP cells (1 × 106) were incubated for 30 min with 1 μM FITC-labeled preS1 peptide (preS1-FITC) and then free peptide were washed out with PBS supplemented with 0.5% BSA and 0.1% sodium azide. Positive signal was analyzed on Cell Sorter 8000 (SONY).

### HBV εRNA preparation

Dr. A. Takaoka (Hokkaido university) kindly provided the ε-stem template of HBV [47]. To generate HBV-5′ end of HBV pgRNA (or epsilon (ε-stem RNA), we used 100 ng of the annealed DNA oligonucleotides (Supplementary Table S1) as template for *in vitro* transcription under the control of the T7 promoter with MEGAscript (Ambion) as described previously [47].

### Expression profile analysis

All microarray experiments and their data were analyzed according to MIAME guidelines. We identified the genes induced by type I IFN from two GEO Datasets, GSE45365 and GSE32137. From the datasets, we selected seven genes, which contain specific functional domains and whose function in the field of innate immunity remains unknown (Supplementary Table S1). The IFN-inducible properties of these genes were confirmed in our laboratory using mouse dendritic cells (http://www.ncbi.nlm.nih.gov/geo/query/acc.cgi?acc=GSE75690).

### Cell transfection

Cells in each well of the 24-well-plate were transfected with 1 μg of plasmid using Lipofectamine 2000 (Life technologies) according to the manufacturer's directions. Transfected cells were harvested at the indicated time points.

### Immunofluorescence

The Huh7 cells were transfected with the plasmids expressing ISG20 and HBV proteins for 48 h and followed by fixation with 2% paraformaldehyde and permeabilization of the cell membrane with 0.1% Triton X-100. Cells were then immunostained with anti-FLAG or anti-HBc antibodies and the bound antibodies were visualized by Alexa Fluor 488 goat anti-mouse IgG [48]. Nuclei were counterstained with DAPI. Cell were imaged with a Nikon fluorescence microscope and photographed with a charge-coupled device camera.

### ELISA of HBs antigen

Concentration of HBsAg in the mice serum or culture supernatant was quantified by sandwich ELISA using commercial ELISA kits following the manufacturer's protocol (XpressBio, USA). The reporting unit was calculated based on O.D 450 nm and extrapolated into the standard curve at ng/mL [10].

### Quantitative RT-PCR (qPCR)

Dnase-I-treated total cellular RNA was used to generate cDNA by SuperScript III Reverse Transcriptase (Life Technologies). Real-time PCR was performed with SYBR Green Master (Roche) and the LightCycler 480 System (Roche) by using HBV, Isg20, and some other gene-specific primers (Supplementary Table S1). The gene expression data were normalized with reference to the level of GAPDH or β-actin in the same samples.

### Statistical analysis

The statistical significance of the obtained data in this study was analyzed using a two tail unpaired *t* test and *p* < 0.05 was regarded as statistically significant.

## SUPPLEMENTARY MATERIALS FIGURE AND TABLES



## Abbreviations

BMDC: marrow-derived dendritic cells
cccDNA: covalently closed circular DNA
CHB: chronic hepatitis B
DC: dendritic cells
HBV: hepatitis B virus
IFN: interferon
IFNAR: IFN-alpha receptor
IRF: interferon-regulatory factor
ISG: interferon-inducible gene
MAVS: mitochondrial antiviral signaling
TICAM-1: TIR-containing adaptor molecule-1
TLR: toll-like receptor
pgRNA: pre-genomic RNA;
