# Supplementary material for: Interferon-stimulated gene of 20 kDa protein (ISG20) degrades RNA of hepatitis B virus to impede the replication of HBV in vitro and in vivo
Source: Oncotarget. 2016 Sep 8;7(42):68179–93. doi: 10.18632/oncotarget.11907 (PMC5356548; doi:10.18632/oncotarget.11907)
Supplement: Supplementary file 1 [file oncotarget-07-68179-s001.pdf]

Interferon-stimulated gene of 20 kDa protein (ISG20) degrades RNA of hepatitis B virus to impede the replication of HBV *in vitro* and *in vivo*

Supplementary Materials

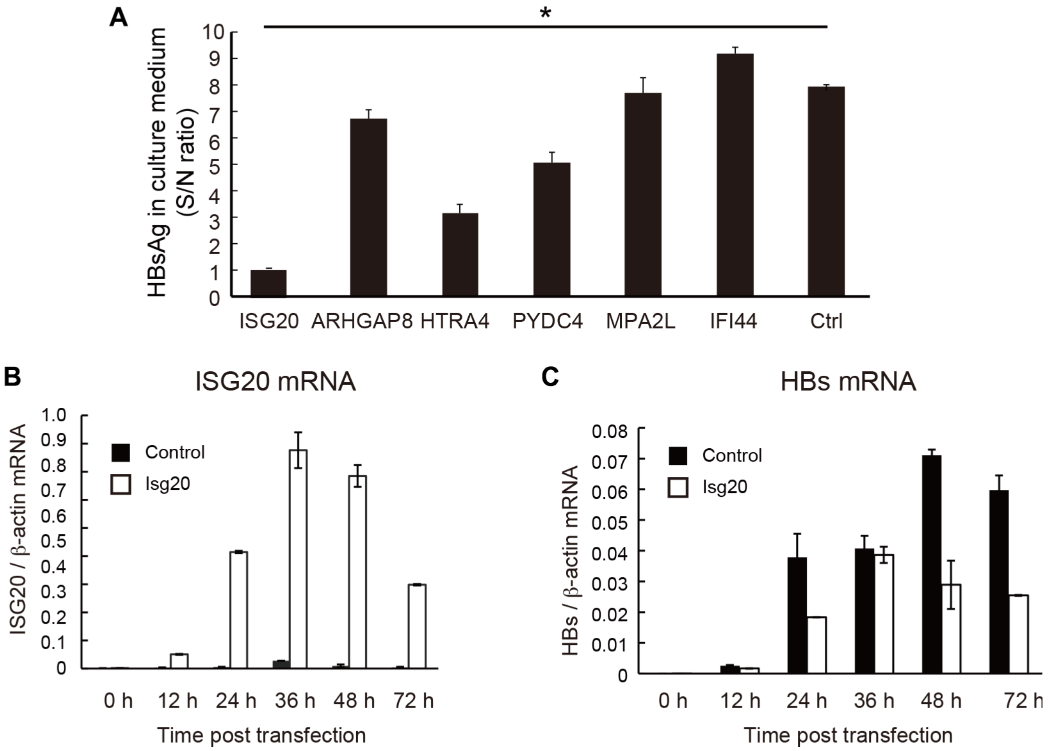

Supplementary Figure S1: Importance of IFN-stimulated genes in anti-HBV response. (**A**) ISG20 expression suppresses HBsAg production in human hepatoma cells. The 6 candidates of interferon-stimulated genes were co-transfected with the pTER1.4xHBV into Huh7 cells to screen for the anti-HBV activity. HBsAg in the culture supernatant after 72 h post transfection was analyzed with ELISA. Data represent 3 independent experiments. *$p < 0.05$. (**B**) and (**C**) HepG2 cells in the 24-well plates were co-transfected with 0.5 µg of Isg20 or control plasmid and pTER1.4xHBV. Total RNA were isolated at the indicated time point post transfection and the expression of Isg20 and HBs mRNA was determined respectively with qPCR.

Supplementary Table S1: Primers used in this study

HBV
F 5′- TTCCTCTTCATCCTGCTGCT -3′
R 5′- GTCCCGTGCTGGTAGTTGAT -3′

Mouse IFN-β
F 5′ -CCAGCTCCAAGAAAGGACGA -3′
R 5′- CGCCCTGTAGGTGAGGTTAT-3′

Mouse IFN-α2
F 5′- TACTCAGCAGACCTTGAACC-3′
R 5′- GGTACACAGTGATCCTGTGG-3′

Mouse IFN-ɤ
F 5′-GATATCTGGAAGGAACTGGCAAAAG -3′
R 5′- AGAGATAATCTGGCTCGGCTCTGCAGGAT -3′

Mouse β-actin
F 5′- TTTGCAGCTCCTTCGTTGC-3′
R 5′- TCGTCATCCATGGCGAACT-3′

Mouse ISG20
F 5′- CAATGCCCTGAAGGAGGATA-3′
R 5′- TGTAGCAGGCGCTTACACAG-3′

Human ISG20
F 5′- TAGCCGCTCATGTCCTCTTT-3′
R 5′-TGAGGGAGAGATCACCGATT -3′

Supplementary Table S2: Database analysis of IFNAR–mediated induction of ISGS

GSE number	GSE32137	ID	*P*.Value	Gene.symbol	Gene.title
Cells	CD11bDC	1423555_a_at	1.59E-05	Ifi44	interferon-induced protein 44
stimulation	MCMV	1437636_at	1.24E-04	Pydc4	pyrin domain containing 4
comparison	between WT and IFNARKO	1419569_a_at	1.79E-03	Isg20	interferon-stimulated protein
		1437273_at	1.17E-02	Htra4	HtrA serine peptidase 4
		1451320_at	1.45E-02	Arhgap8	Rho GTPase activating protein 8
		1438676_at	2.02E-02	Gbp6	guanylate binding protein 6
		1456288_at	2.25E-02	Slfn5	schlafen 5

GSE number	GSE45365	ID	*P*.Value	Gene.symbol	Gene.title
Cells	Airway epithelial cells	A_52_P189772	1.19E-08	Gbp6	guanylate binding protein 6
stimulation	Influenza Virus	A_51_P487690	6.94E-08	Ifi44	interferon-induced protein 44
comparison	between WT and IFNARKO	A_52_P448253	1.01E-06	Isg20	interferon-stimulated protein
		A_52_P483799	1.59E-06	Pydc4	pyrin domain containing 4
		A_51_P364694	5.64E-06	Slfn5	schlafen 5
		A_51_P417074	1.09E-05	Arhgap8	Rho GTPase activating protein 8
		A_52_P207314	1.58E-01	Htra4	HtrA serine peptidase 4

GSE number	GSE75690	ID	*P*.Value	Gene.symbol	Gene.title
Cells	CD8+ DC	1458458_at	9.44E-08	Slfn5	schlafen 5
stimulation	poly I:C	1444405_at	1.40E-06	Pydc4	pyrin domain containing 4
comparison	between control Ab and anti-IFNAR Ab	1423555_a_at	1.76E-05	Ifi44	interferon-induced protein 44
		1438676_at	6.71E-05	Gbp6	guanylate binding protein 6
		1419569_a_at	9.33E-05	Isg20	interferon-stimulated protein
		1451320_at	3.71E-03	Arhgap8	Rho GTPase activating protein 8
		1437273_at	9.31E-02	Htra4	HtrA serine peptidase 4
